# Nonpharmaceutical Interventions for Pandemic Influenza, International Measures

**DOI:** 10.3201/eid1201.051370

**Published:** 2006-01

**Authors:** World Health Organization Writing Group

**Keywords:** influenza, World Health Organization, quarantine

## Abstract

Since global availability of vaccine and antiviral agents against influenza caused by novel human subtypes is insufficient, the World Health Organization (WHO) recommends nonpharmaceutical public health interventions to contain infection, delay spread, and reduce the impact of pandemic disease. Virus transmission characteristics will not be completely known in advance, but difficulties in influenza control typically include peak infectivity early in illness, a short interval between cases, and to a lesser extent, transmission from persons with incubating or asymptomatic infection. Screening and quarantining entering travelers at international borders did not substantially delay virus introduction in past pandemics, except in some island countries, and will likely be even less effective in the modern era. Instead, WHO recommends providing information to international travelers and possibly screening travelers departing countries with transmissible human infection. The principal focus of interventions against pandemic influenza spread should be at national and community levels rather than international borders.

## Abstract 2

Closing international borders was usually ineffective in past pandemics and would be less effective today.

Pandemic preparedness ideally would include pharmaceutical countermeasures (vaccine and antiviral drugs), but for the foreseeable future, such measures will not be available for the global population of >6 billion (*1*). Thus, in 2005, after consultations with experts, the World Health Organization (WHO) recommended nonpharmaceutical public health interventions in its updated global influenza preparedness plan (*2*). The recommendations are intended as guidance, not as formal WHO advice (*3*). Such interventions, designed to reduce exposure of susceptible persons to an infectious agent, were commonly used for infection control in previous centuries. This report (part 1) and a companion article (part 2 [4]) summarize the scientific data, historic experience, and contemporary observations that make up the limited evidence base for these interventions as applied to influenza. Part 1 summarizes the relevant transmission characteristics of influenza and the basis for interventions to prevent spread from 1 country to another; part 2 summarizes the basis for measures within countries at the national and community levels. Both parts are designed to be read in conjunction with WHO recommendations (*2**,**3*).

Nonpharmaceutical interventions outside of healthcare settings focus on measures to 1) limit international spread of the virus (e.g., travel screening and restrictions); 2) reduce spread within national and local populations (e.g., isolation and treatment of ill persons; monitoring and possible quarantine of exposed persons; and social distancing measures, such as cancellation of mass gatherings and closure of schools); 3) reduce an individual person's risk for infection (e.g., hand hygiene); and 4) communicate risk to the public. We discuss the first category; categories 2 and 3 are addressed in part 2. We do not address infection control measures for patient care or risk communication.

## Transmission Characteristics of Influenza Viruses

Most information on transmission of influenza viruses is based on older experimental studies, inference from observations during outbreaks, and studies with other objectives, especially the assessment of vaccine or drug efficacy. These sources have substantial limitations: investigations often used different methods, involved small numbers of persons, and reflected the behavior of influenza A and B viruses in seasonal rather than pandemic settings (the level of preexisting immunity in populations is substantially higher in seasonal epidemics). For this reason, data from young children, who presumably lack prior exposure and therefore immunity to influenza, may better reflect illness and viral shedding patterns of pandemic disease. The "infectiousness" of patients is virtually always inferred on the basis of viral shedding from the upper respiratory tract rather than from directly observed transmission, but the relationship between nasopharyngeal shedding and transmission is uncertain and could vary. Detailed studies of lower respiratory tract virus loads, particularly relevant to small-particle aerosol transmission during coughing and sneezing, are not available. In many studies, the preexisting influenza antibody status of study participants is not reported, even though this factor is critical in influencing illness and viral shedding patterns. In controlled studies, in which susceptible study participants are typically screened for preexisting influenza antibody by hemagglutination inhibition assays to the challenge virus, the routes of infection and the challenge virus can differ. Other factors that differ among studies are the age and preexisting medical conditions of study participants and the timing of specimen collections for virus testing.

### Viral Shedding and Transmission by Persons with Symptoms

In otherwise healthy adults with influenza infection, viral shedding 24–48 h before illness onset has been detected but generally at much lower titers than during the symptomatic period (Table A1). Titers of infectious virus peak during the first 24–72 h of illness (10^3^–10^7^ 50% tissue culture infective dose [TCID_50_]/mL nasopharyngeal wash) and decline within several days, with titers usually low or undetectable by day 5. Shedding in highly immunocompromised persons may last weeks to months. Compared with adults, children can shed virus earlier before illness begins and for longer periods once illness starts. As in adults, peak shedding in children occurs during the first 1–3 days of illness, but absolute levels may be higher than those in adults. In 1 report, at least 4 illnesses (8% of the total) in children were associated with presymptomatic shedding that began 6, 4, 3, and 3 days, respectively, before illness onset (*5*). The median duration of virus detection is typically 7–8 days after illness onset, but shedding for up to 21 days has been recorded. In 1 study, virus was shed by 10% of children on days 8–11, by 5% on days 12–15, and by 0% on days 16–19 (*6*). Infants with infection requiring hospitalization may shed virus longer. In both adults and children, shedding does not usually continue once illness has resolved. Serologic testing indicates that ≈30%–50% of seasonal influenza infections may not result in illness.

### Viral Shedding and Transmission by Infected Persons without Symptoms

During the incubation period, persons with presymptomatic influenza infection shed virus at lower titers than persons with symptoms (Table A1); however, the infectiousness of those with presymptomatic infection has not been studied. Apparently the only published report implicating transmission during the incubation period involves a group of adults in New Zealand in 1991. Of 26 adults who bagged fertilizer for 8 h, influenzalike illness (fever, headache, sore throat, myalgia, respiratory symptoms) developed in 16 and mild, "cold-like" illnesses developed in 3 persons within 24 to 48 h after working with the fertilizer. A person considered to be the probable index patient had felt unwell during work, although he did not have respiratory symptoms; an influenzalike illness began to develop 6 h after he finished work. Influenza A virus H1N1 was isolated from 2 symptomatic persons; whether these included the suspected index patient and whether that person transmitted infection during an incubation period or the cluster resulted from community exposure are unknown. The group shared drinking bottles and worked in a dusty environment, both of which could have facilitated transmission (*7*).

### Large-Droplet and Aerosol Respiratory Transmission

Animal studies and most influenza outbreaks among humans suggest that virus-laden large droplets (particles >5 mm in diameter) generated when infected persons cough or sneeze are the predominant mechanism of influenza virus transmission (*8*). However, evidence for aerosol spread (especially in unventilated conditions) is available (*9*). Although a direct comparison has not been made, experimental studies suggest that the infectious dose for humans exposed by aerosol is lower than that seen with experimental nasopharyngeal instillation (*10*). The precise proportion of infections transmitted by large droplets versus aerosols is difficult to assess and likely depends on the setting but is relevant when developing recommendations on mask use. Data do not exist to quantify the relative efficacy of surgical masks versus respirators in preventing influenza infections in exposed persons, but surgical masks should protect against large droplets, believed to be the major mode of transmission (*8*).

### Transmission by Contaminated Hands, Other Surfaces, or Fomites

Transmission of influenza viruses by contaminated hands, other surfaces, or fomites has not been extensively documented but is believed to occur. In a nursing home outbreak in Hawaii, an investigation concluded that transmission of oral secretions from patient to patient by staff who were not gloved best explained the outbreak (*11*). In an environmental survival study, influenza A virus placed on hard, nonporous surfaces (steel and plastic) could be cultured from the surfaces at diminishing titer for <24 to 48 h and from cloth, paper, and tissues for <8 to 12 h at conditions of 35% to 40% humidity and a temperature of 28°C (*12*). Higher humidity shortened virus survival. Virus on nonporous surfaces could be transferred to hands 24 h after the surface was contaminated, while tissues could transfer virus to hands for 15 min after the tissue was contaminated. On hands, virus concentration fell by 100- to 1,000-fold within 5 min after transfer. The authors concluded that transmitting infection from the surfaces tested would require a high titer of virus (10^5.0^ TCID_50_/mL) on the surface; such titers can be found in nasal secretions at an early stage of illness.

### Incubation Period and Infectiousness

The incubation period for influenza averages 2 days (range 1–4 days), and the serial interval (the mean interval between onset of illness in 2 successive patients in a chain of transmission) is 2–4 days. Also, viral excretion peaks early in illness. These factors enable influenza to spread rapidly through communities. By contrast, severe acute respiratory syndrome (SARS) has a serial interval of 8 to 10 days, and peak infectivity does not occur until week 2 of illness, which allows more time to effectively implement isolation and quarantine measures (*13*). The basic reproduction number (*R*_0_, the mean number of secondary cases generated by 1 infected person in a fully susceptible population) of the 1918 pandemic influenza subtype has recently been re-estimated as ≈2–3 (*14*) and 1.8 (*15*), comparable to that of the SARS-associated coronavirus (SARS-CoV) (*R*_0_ 2–4) (*13*).

### Amplifying Groups and Settings

Children in preschool and school-age groups are frequently observed to amplify transmission (*16*), although any group living in close proximity can do so, and outbreaks are observed in institutions involving persons of all ages (*11*). Although transmission may be amplified at mass gatherings (e.g., theaters, sports events), documentation is scarce.

## Slowing or Preventing International Spread of Pandemic Influenza

### Experience from Earlier Pandemics

#### 1918 Experience with Quarantine Enacted by Islands

In the 1918 pandemic, some island countries enacted maritime quarantines that appear to have delayed or prevented the introduction of pandemic influenza. Maritime quarantines were facilitated because ships had often been at sea for an extended period, reducing the likelihood of ongoing onboard infection at the time of arrival in port. Also, authorities could require ships to anchor in harbors or at quarantine stations on offshore islands, thus minimizing contact with persons on shore.

In October 1918, Australia began to quarantine arriving ships upon which a case of influenza had occurred during the voyage; the duration of quarantine was determined on the basis of the date of the most recent case. Quarantine was also applied for 7 days, even if no cases were reported, to vessels arriving from New Zealand and South Africa because of severe epidemic disease in those areas and from certain Pacific Islands with which communication was limited. Persons in quarantine had their temperature measured at least once daily, and those with an oral temperature >99°F (37.2°C) were isolated at hospitals for observation. Measures taken by hospital staff to avoid infection included the use of masks and other "routine precautions taken at isolation hospitals." Reportedly, no direct evidence of escape of infection from any vessel to the shore occurred.

From October 1918 through May 1919, a total of 79 "infected vessels" containing 2,795 patients, 48,072 passengers, and 10,456 crew and 149 "uninfected vessels" containing 7,075 passengers and 7,941 crew arrived at Australian ports (*17**,**18*). The first cases of pandemic influenza in Australia were reported in January 1919, suggesting that these measures delayed entry of the disease for ≈3 months. Although the national quarantine director believed that pandemic influenza had entered Australia before quarantine was established, this belief was not documented, and other reports indicate that some ships' officers and soldiers returning to Australia from Europe had concealed illness to avoid protracted quarantine (*18*). When the infection did emerge in Australia, case-fatality rates were lower than those in many places affected earlier.

According to a report from the New South Wales Department of Public Health, ships with ill passengers arrived regularly at Sydney (the state capital) from October 1918 to January 1919. Of 326 passengers or crew treated at the quarantine hospital, 49 died. Recovered patients and contacts emerging from quarantine were released into the general population and monitored by health officials for a few days to a few weeks. Two cases were in nurses who had contracted influenza while caring for patients at the quarantine hospital. "In no case did any suspicion arise that such persons had spread influenza among those with whom they had come in contact" (*19*). The first cases of influenza in New South Wales were in soldiers who arrived overland by train from the port city of Melbourne, Victoria, where recent cases were known to have occurred but were not promptly disclosed by the authorities (*19*).

In 1918, the island of Madagascar, then a French colony, also implemented a "rigorous quarantine" and did not report cases of influenza until April 1919. In contrast, nearby coastal regions of eastern and southern Africa reported cases beginning in September to December 1918. Contact between Madagascar and South Africa, where the disease was epidemic, was limited to a single coastal steamboat (*20**,**21*). In the Pacific, American Samoa implemented quarantine measures and was spared infection, while nearby islands were severely affected (*22*). The French colony of New Caledonia was spared infection by requiring ships to remain in quarantine at their ports of departure, a form of "exit screening," discussed below (*23*).

#### Other Quarantine Experiences

On the African mainland, quarantine was enacted in 1918 in some port cities in, for example, Liberia, Gabon, and Ghana (formerly known as the Gold Coast). Details generally are unavailable, but, on the whole, even though entry may have been delayed by some weeks, the experience was less successful than that of islands that enacted quarantine. Disease arrived from inland routes and, according to 1 report, quarantine of a ship in Accra, Ghana, known in advance to be carrying persons with influenza was not successful; disease spread to dock workers and subsequently entered the country (*21**,**24*).

In 1918, closing roads at the northern land border of Ghana was not feasible because of the volume of trade and the probability that police barriers would be evaded. An attempt was nevertheless made to close roads at the border town of Tumu, but authorities concluded that "a handful of constables could not stop the epidemic and the effort was soon abandoned" (*24*). In Canada and Australia, substantial measures, including police checkpoints and interruption of road and rail traffic, did not prevent or appear to delay the spread of infection between Canadian provinces or Australian states (*4**,**18*).

A WHO expert consultation on the 1957 influenza pandemic summarized the effect of quarantine measures at international borders as follows. Onset in Israel was delayed by 2 months in comparison to neighboring countries, attributed to absence of international travel with neighboring countries (for political, not quarantine reasons). In South Africa, "some delay" occurred from restrictions on ships arriving at ports, but the evidence was "less convincing." Elsewhere, "no effect was detected. It seems that if such measures are to be effective, they must be very severe…. a high price to pay for a few additional weeks freedom from the disease" (*25*).

### Experience from Contemporary SARS and Influenza Outbreaks

In modern times, the most extensive use of nonpharmaceutical public health interventions to contain a transmissible respiratory viral infection occurred during the SARS epidemic of 2003. Some lessons learned from that experience may be applicable to influenza, although important differences exist between the epidemiologic parameters of influenza virus and SARS-CoV. The most notable of these are that influenza has a serial interval of 2 to 4 days and infectivity is maximal early in illness, whereas for SARS the serial interval is 8–10 days and infectivity peaks during week 2 of illness. These factors allow little time for instituting the isolation and quarantine interventions that were essential in controlling SARS.

#### Entry Screening of Air-travel Passengers during 2003 SARS Outbreak

In the 2003 SARS experience, data from 4 Asian locations and Canada indicated that body temperature–sensing devices did not detect anyone with SARS among >35 million entering travelers screened. Administration of health declarations (a questionnaire completed by the traveler to report health information, e.g., symptoms and history of exposure) to >45 million entering travelers detected 4 SARS cases. At least 31 million health alert notices were distributed to entering international travelers in several countries, but follow-up information is limited. Mainland China reported the distribution of 450,000 notices and detection of 4 SARS cases possibly linked to the notices. Thailand reported printing 1 million notices and detecting 24 cases directly linked to them (*26*). The 5 persons with SARS who entered Canada did not have signs or symptoms at international airports; Canadian authorities concluded that border screening for SARS was insensitive and not cost-effective and that surveillance allowing for early detection of imported cases was preferable (*27*).

The possible effect of entry screening for pandemic influenza has been estimated for the United Kingdom, with the assumption that exit screening is in place at international airports in countries with pandemic influenza. A mean of 9% of persons infected by influenza who were asymptomatic on departure would be estimated to develop influenza symptoms en route to the United Kingdom; the percentage would be higher during longer flights. Symptoms would develop in an estimated mean of 17% (range 12%–23%) of infected persons traveling from Asian cities. Airplanes that arrive daily at 12 airports in the United Kingdom from the Far East have >12,000 seats; entry screening would fail to detect ≈83% of infected persons (*28*). Travelers arriving on connecting flights were not considered. In Taiwan during the 2003 SARS outbreak, 80,813 incoming air-travel passengers from affected areas were quarantined; 21 (0.03%) were diagnosed with suspected or probable SARS. None of these 21 cases had been detected by entry screening (*26**,**29*). Another modeling study from the UK Health Protection Agency suggests that reduction of air travel to and from affected areas, if implemented, must be almost total and nearly instantaneous to delay pandemic spread significantly (B. Cooper, pers. comm.).

#### Exit Screening of Travelers during SARS Outbreak

After WHO recommended exit screening of international travelers departing from affected areas on March 27, 2003, no additional spread of SARS through air travel was documented from countries with exit screening. This finding may reflect a deterrence effect, a generally low incidence of SARS cases, or both. Combined data from several countries indicate 1 case detected among 1.8 million departing passengers completing health questionnaires and no cases among 7 million persons who underwent thermal scanning on departure (*26*).

#### Measures To Limit Influenza Virus Transmission on Conveyances

Influenza has been transmitted on airplanes (*30*) and ships (*31*). In 1 cluster, influenzalike illness developed in 72% of passengers seated in an airplane that was on the ground for 3 h without ventilation and that held a person with symptomatic influenza (*9*). On a 75-seat aircraft, 15 passengers traveling with an influenza-infected person became ill. All 15 persons were seated within 5 rows of the index patient, and 9 were seated within 2 rows (*32*).

In a review of the Australian experience with pandemic influenza aboard ships in 1918 to 1919, a "Daily thermometer parade and removal of any person febrile or reporting sick (was) most thoroughly and efficiently carried out" (*17*). Despite these measures, examples were given of 3 ships with 89%, 46%, and 30%, respectively, of those onboard who were ill, which led to the "conclusion that neither inhalation, inoculation, nor isolation of the sick would stop an epidemic. . . . No administrative measure was successful in modifying the time factor of a shipboard epidemic, although there is some reason for believing that the measures employed were, by their combined influence, successful in reducing the potential volume of actual cases" (*17*).

Influenza outbreaks have been reported on cruise ships during international voyages (*31*). A large summertime outbreak involved both international travelers and crew during 3 cruises of 1 ship. Control measures included surveillance, isolation of ill crew, immunization of the crew, and use of antiviral drugs for treatment and prophylaxis of crew and passengers (*31**,**33*).

During the 2003 SARS outbreak, the disease was transmitted on and spread internationally via aircraft. The most extensive investigation included 3 flights on which an index passenger had SARS; on 1 of these flights, 22 (18.3%) of 120 other passengers and crew became infected. A higher risk was noted for passengers seated near the index patient, but most passengers who became infected were seated farther away, even though their individual risk was lower (*34*). In most other investigations, no transmissions were documented, although the investigations were limited (*26*).

## Discussion

The effectiveness of nonpharmaceutical public health interventions in affecting the spread of pandemic influenza depends on transmission characteristics of the virus. If a substantial proportion of transmission occurs during the incubation period or during asymptomatic infection, the population impact of health screening and case-patient isolation will be diminished. The age distribution of patients is also important: if children play a central role in initial community transmission, school closure would likely be more effective. Since a new pandemic subtype might have different transmission characteristics than previous subtypes, these characteristics and associated illness patterns must be assessed in the field as soon as human-to-human transmission begins. Monitoring over time is also needed to assess possible changes as the virus becomes more adapted to human hosts.

WHO has developed recommendations to provide guidance until transmission characteristics can be determined. The recommendations are based on limited information, including virologic data from seasonal epidemics and volunteer studies rather than pandemics, in which shedding and transmission may be more intense and prolonged because of lack of population immunity. These data indicate that influenza viral shedding in the upper respiratory tract (and presumably also infectiousness) is correlated with fever and the severity of respiratory symptoms in both adults and children. The importance of transmission from infected persons during the incubation period or from persons with asymptomatic infection is uncertain but appears to be substantially less than from symptomatic persons. The principal difficulties in using nonpharmaceutical interventions to reduce influenza transmission among humans include the peak infectivity early in illness and the short incubation period, which both result in a short serial interval between related cases. Recent reports suggest that the 1918 virus may have been less transmissible than previously thought (*R*_0_ 1.8–3), although whether public health interventions in 1918 might have affected these estimates is uncertain. If a novel human influenza subtype behaves in a manner similar to the pandemic virus of 1918–1919, available information supports the use of nonpharmaceutical interventions to delay or contain transmission during WHO phases 4 and 5 (limited human-to-human transmission) and use of different interventions to reduce the impact in phase 6 (pandemic phase) (*2**,**3*).

At the international level, experience in past influenza pandemics indicates that screening and quarantine of entering travelers at international borders did not substantially delay introduction, except in some island countries. Similar policies, even if they could be implemented in time and regardless of expense, would doubtfully be more effective in the modern era of extensive international air travel. WHO instead recommends that travelers receive health alert notices, although entry screening may be considered when the host country suspects that exit screening at the traveler's point of embarkation is suboptimal; in geographically isolated, infection-free areas (e.g., islands); and where a host country's internal surveillance capacity is limited (*2*).

WHO recommends consideration of exit screening by health declaration and temperature measurement for international travelers departing countries with human infection at phases 4, 5, and 6. Exit screening in affected countries is a better use of global resources: fewer persons would need to be screened, the positive predictive value for ill persons detected would be higher, and transmission on conveyances, such as aircraft, would be reduced. Exit screening is disruptive and costly, however, and will not be fully efficient as influenza viruses can be carried by asymptomatic persons who will escape detection during screening (*2**,**3*). As was true for SARS, the principal focus of WHO-recommended nonpharmaceutical interventions is not at international borders but at national and community levels (*4*).

## Appendix

**Table A1:** Presymptomatic or asymptomatic influenza viral shedding and relationship of shedding with symptoms, as determined in representative studies

First author	Nature of study	Pertinent results	Pertinent conclusions
Frank (*35*)	Surveillance in families, Houston, Texas, 1975–1979	In a study of 50 influenza A illnesses in 41 children <4 years of age, presymptomatic viral shedding was noted in 4 (8%) from whom positive samples were obtained 6, 4, 3, and 3 days, respectively, before symptom onset. Samples were not cultured for most children before symptom onset. Peak shedding occurred during week 1 of illness, when 73% of cultures were positive; 10% were positive on days 8–11, 5% on days 12–15, and 0% days 16–19. Illness duration and shedding were not correlated.	In children, at least 8% of illnesses in a seasonal outbreak were associated with presymptomatic shedding, which was noted up to 6 days before illness; 5% continued to shed at the end of week 2. Peak rates of shedding correlated with symptoms.
Hall (*36*)	Children with respiratory illness seeking medical care during influenza outbreaks, Rochester, New York, 1977 and 1986	In an influenza B study of 43 children (mean age 8 y), 74% had typical influenzalike illness, 7% had afebrile upper respiratory infections, and 19% had croup. Peak virus titers occurred on day 1 of illness, and 93% of children shed virus on days 1–3, 75% on day 4, and 30% on day 6. Severe illness and prolonged fever were associated with higher virus titers, and a trend (p<0.07) for younger children to shed higher-titer virus occurred. In an influenza A study, symptoms and the highest viral shedding occurred early in illness; 30% of patients still shed at day 7.	Peak shedding early in illness, correlated with symptoms and fever.
Davis (*37*)	Surveillance in families, Washington, DC area, 1951–1956	Influenza A virus was recovered from 0/4 and 2/3 throat swab samples obtained 3–5 and 1–2 days, respectively, before symptoms, compared with 75 (59%) of 127 swab specimens collected within 3 days after onset of illness.	Shedding at low levels occurred 1–2 days before symptoms
Foy (*38*)	Surveillance in families, Seattle, Washington, 1984	12/37 persons, all >12 y of age, from whom influenza B virus was isolated were asymptomatic. Virus cultures from asymptomatic persons required longer incubation time than those from symptomatic persons to become positive (12.7 vs. 8.7 days, p<0.007), suggesting low titers of virus in these specimens. The study design did not permit assessment of whether asymptomatic persons transmitted influenza.	Asymptomatic shedding at a low titer may be common during a seasonal outbreak.
Murphy (*39*)	In the control arm of a study, 7 adult volunteers were nasally infected with wild-type H3N2 virus	Peak virus shedding occurred 2 days after nasal inoculation, and shedding ceased by day 6. Titer of virus shed and daily fever score (reflects height and duration of fever) were strongly correlated (p<0.001). 1/7 study participants had mild symptoms without fever and shed less virus.	Fever score and quantity of virus shed were strongly correlated.
Couch (*40*)	In a vaccine study, 29 adults received vaccines, and 11 controls received saline. All were nasally infected with H3N2	The vaccinated group had a reduced rate and severity of illness. Among controls (without specific antibody), titer of virus shed and daily severity of illness scores (p<0.001) were strongly correlated. In the combined group, peak viral shedding occurred 3 days after infection. Virus was shed a mean of 5.7 days. 6/29 vaccine recipients and 5/11 controls shed low levels of virus but did not become ill.	Severity of illness and quantity of virus shed were strongly correlated; a substantial rate of asymptomatic shedding occurred at low titer.
Khakpour (*41*)	Daily cultures of 29 adult prisoners after natural exposure to influenza, Iran, 1968	Virus was isolated from 5/29 contacts. One person had influenza isolated from a throat washing and blood, which is rare, 12 h before symptoms. Four persons had virus isolated from throat washings but never had symptoms; acute antibody titer was high in 2. Virus titrations were not performed.	Presymptomatic and asymptomatic shedding can occur.
Philip (*42*)	Surveillance in families, Washington, DC area, 1952–1955	In households with proven influenza illness, influenza A was isolated from 8 (38%) of 21 contacts with acute afebrile respiratory illness and 2 (5%) of 40 healthy contacts. Influenza B virus was isolated from 2 (11%) of 19 contacts with acute afebrile respiratory illness and 0 of 47 contacts. Age distribution of culture-positive contacts was not reported.	Symptomatic household contacts commonly shed influenza A virus. Asymptomatic shedding of influenza A and B viruses was uncommon.
Monto (*43*)	Surveillance in families, Tecumseh, Michigan, 1976–1981	During seasonal epidemics among persons with serologically proven influenza A (H3N2) infection, at least 15%–25% were ill. Among persons with serologically proven influenza B, at least 19%–34% were ill. Among persons with febrile respiratory illness, influenza virus was isolated from 19.7%.	≈60% of seasonal infections were asymptomatic. (This includes persons with partial immunity due to infection with the same subtype as in previous years; the percentage of asymptomatic infections would be lower in a pandemic.) Viral shedding in asymptomatic persons is likely to be quite low.
Hayden (*44*)	19 volunteers infected with influenza A H1N1	All study participants were symptomatic, and symptom scores peaked on day 2 and returned to normal by day 8. Virus titers correlated with symptoms.	Viral shedding was correlated with symptoms.
